# Subclinical Lesions of the Primary Clinical Target Volume Margin in Esophageal Squamous Cell Carcinoma and Association With FDG PET/CT

**DOI:** 10.3389/fonc.2019.00336

**Published:** 2019-04-30

**Authors:** Dali Han, Yinping Yuan, Jie Chai, Guifang Zhang, Lili Wang, Aijun Ren, Pingping Song, Zheng Fu, Jinming Yu

**Affiliations:** ^1^Department of Radiation Oncology, Shandong University Affiliated Shandong Cancer Hospital and Institute, Jinan, China; ^2^Key Laboratory of Radiation Oncology of Shandong Province, Jinan, China; ^3^Department of Pathology, Shandong University Affiliated Shandong Cancer Hospital and Institute, Jinan, China; ^4^Department of General Surgery, Shandong University Affiliated Shandong Cancer Hospital and Institute, Jinan, China; ^5^Department of Oncology, Shandong University Affiliated Shandong Cancer Hospital and Institute, Jinan, China; ^6^Department of Oncology, Yucheng City People's Hospital, Dezhou, China; ^7^Department of Thorax Surgery, Shandong University Affiliated Shandong Cancer Hospital and Institute, Jinan, China; ^8^Department of Nuclear Medicine, Shandong University Affiliated Shandong Cancer Hospital and Institute, Jinan, China

**Keywords:** pathology, subclinical lesion, clinical target volume, esophageal squamous cell carcinoma, ^18^F-fluorodeoxyglucose

## Abstract

**Background and Objectives:** An accurate delineation of the primary clinical target volume (CTVp) in esophageal squamous cell carcinoma (ESCC) significantly affects the outcomes of radiotherapy. However, when basing the CTVp on the primary gross tumor volume, there are no consistent guidelines for the size of the margin. We compared preoperative ^18^F-fluorodeoxyglucose (FDG) PET/CT images and large slices of resected pathological ESCC specimens for evidence and prediction of subclinical lesions. We also investigated associations between the maximum standardized uptake value (SUVmax), metabolic tumor volumes (MTVs), and lesions to improve estimates of the CTVp.

**Methods:**55 patients underwent FDG PET/CT before surgery, and the SUVmax and MTVs were determined. To ensure that the *in situ* distances between the primary and secondary tumors were preserved, the esophageal specimens collected during radical surgery were processed to minimize shrinkage, and subclinical lesions were characterized by pathological examination. A 2-dimensional logistic regression model was used to assess the associations between clinicopathological features and microscopic spread of the lesions.

**Results:** Subclinical lesions in pathological specimens were characterized as direct invasion, multicentric occurrence lesions, intra-mural metastasis, vascular invasion, and perineural invasion in 56.4, 40.0, 30.9, 21.8, and 18.2% of patients, respectively. The mean distances of the subclinical lesions from the primary tumor were 0.79 ± 1.28 cm and 0.87 ± 1.00 cm in the cranial and caudal directions, respectively. Together the SUVmax and MTV values could predict the presence of subclinical lesions that were not detectable in PET/CT images.

**Conclusions:** To cover 94.5% of ESCC subclinical lesions in the CTVp, a 3-cm margin along the cranial-caudal axis should be added to the primary gross tumor volume as defined by FDG-PET/CT, as well as a cutoff SUVmax value of 2.5. Although preoperative FDG PET/CT images may not reveal lesions directly, the SUVmax and MTV measurements together could predict their presence.

## Background

Esophageal carcinoma often occurs as squamous cell carcinoma (ESCC), a highly aggressive malignancy with a poor prognosis worldwide. The incidence rates of ESCC are particularly high in China (1). Most patients with ESCC have locally advanced disease (2, 3). Important treatment strategies for locally advanced ESCC include neoadjuvant chemoradiation and definitive chemoradiation or radiation therapy. However, the overall survival and local control rates remain unsatisfactory—the 2-year survival rate is merely 30–40%, and the local relapse rate may reach up to 50% (4–6).

In ESCC radiotherapy, an accurate delineation of the primary clinical target volume (CTVp) importantly influences the success of the outcome, with higher local regional control, and less toxicity. Wu et al. suggested that CTVp is generally a 3–4 cm superior and inferior expansion of the gross tumor volume (GTV) and a 1 cm radial expansion, according to an expert consensus (7). However, when basing the CTVp on the size of the primary gross tumor, there are no established or standard guidelines for how large the margin should be based on precision data. Countries, institutions and even physicians have different viewpoints on this issue. We reviewed the original literature in ESCC research pathology (8) and found that the CTVp commonly consists of the primary tumor and surrounding secondary lesions, which frequently include direct, vascular and perineural invasion, intra-mural metastasis, and multicentric occurrence lesions.

Currently, there is no technology capable of accurately detecting subclinical tumor lesions, and the optimal CTVp in esophageal cancer remains controversial. To provide a theoretical basis for the clinical determination of the CTVp in esophageal cancer, this study examined the pathology of the longitudinal distribution of subclinical lesions in long resected specimens. For the closest estimate of the length of the primary gross tumor volume, we previously reported a standardized ^18^F-fluorodeoxyglucose (FDG) uptake cutoff value of 2.5 in positron emission tomography (PET)/computed tomography (CT) (9, 10). In this study, we investigated the correspondence between the results of ESCC pathology and FDG PET/CT, as well as pathological results that help determine the most appropriate CTVp in these patients.

## Methods

The study was designed and conducted at Shandong University Affiliated Shandong Cancer Hospital as a prospective study with clinical samples handled *in vitro* (without registration). The Ethics Committee of Shandong University Affiliated Shandong Cancer Hospital and Institute approved the study protocol on 5 March 2011 (Ethics Approval No. SDTHEC201103009). All patients gave their written informed consent for experiments, in accordance with the Code of Ethics of the World Medical Association (Declaration of Helsinki).

### Patients

In general, to meet the basic requirements of model estimation, the sample size *n* should be >30, or *n* ≥ 3(k + 1), where k is the number of independent variables. However, while a sample size of 30 is considered the minimum for a quantitative study, subclinical lesions are usually found in < 10% of ESCC patients (7). Thus, we sought an enrolled sample size of 55, and the final study population was comprised of 55 patients treated between January 2012 and September 2015 at Shandong University Affiliated Shandong Cancer Hospital.

All of the analyzed patients conformed to the following inclusion criteria: histologically proven ESCC, Karnofsky performance status (KPS) score >70, routine pretreatment evaluation, including barium esophagography, gastroscopy, ultrasound evaluation of the neck and abdomen, and pulmonary function testing; radical surgery 3–5 d after a FDG PET/CT scan, no previous chemotherapy or radiotherapy, and no history of a malignant tumor. Patients were excluded from this study if any of the above inclusion criteria were not met.

All eligible patients without distant metastasis, or definite direct invasion of adjacent organs detected on imaging, were scheduled as routine for esophageal resection and extensive regional lymph node dissection. They underwent a standard esophagectomy based on the McKeown method. A 3-field lymph node dissection was also performed, if necessary.

### Imaging Protocols and the Parameters of FDG PET/CT in Patients

The patients were required to fast for 6 h and to rest for 15 min before i.v. injection of 370 MBq (10 mCi) FDG. Patients were also asked to drink 500 mL of water before imaging to stimulate FDG excretion from the renal calyces and subsequent voiding.

The images were acquired 60 min p.i. using a standard clinical PET/CT scanner (Discovery LS; GE Healthcare) on the basis of the clinical situation and with the patient's consent. Emission scans were performed in whole-body mode for 5 min per field of view from head to thigh. Each field covered 14.5 cm at an axial sampling thickness of 4.25 mm/slice. The PET/CT system was used for 4-slice helical CT acquisition, followed by a full-ring dedicated PET scan of the same axial range. The CT component was operated with an X-ray tube peak voltage of 120 keV at 90 mA, 6:1 pitch, 4.25 mm slice thickness and a rotational speed of 0.8 s/ rotation. Both the PET and the CT scans were acquired during normal tidal breathing.

PET images were reconstructed with CT-derived attenuation correction by iterative reconstruction using ordered-subset expectation maximization (OSEM). The images (attenuation-corrected PET, CT, and fused PET/CT) were reviewed in the axial, coronal and sagittal planes on a dedicated workstation (Xeleris; GE Healthcare) computer monitors, as was a cine display of the maximum intensity projections of the PET data.

The PET images were visually inspected, and the maximum standardized uptake value (SUVmax) was determined from circular regions of interest drawn around the lesion in all consecutive image slices covering the entire lesion. The metabolic tumor volume (MTV) was delineated in FDG PET/CT images using a fixed SUV threshold value of 2.5 (8, 9). The imaging area covered the metabolically active area. The boundary was outlined layer by layer, and to calculate the volume the area of each layer was multiplied by the layer thickness. Then, the volumes of each layer were combined to calculate the MTV. If the SUVmax was less than the standard value, the MTV was calculated as a single voxel with volume of 0.1 mL.

Due to PET partial volume effects, the determination of the MTV were excluded in the cavity regions. All PET images were further reviewed by two physicians who were experienced in nuclear medicine.

### Preparation of Large Pathological Slices

**Figure 1:**
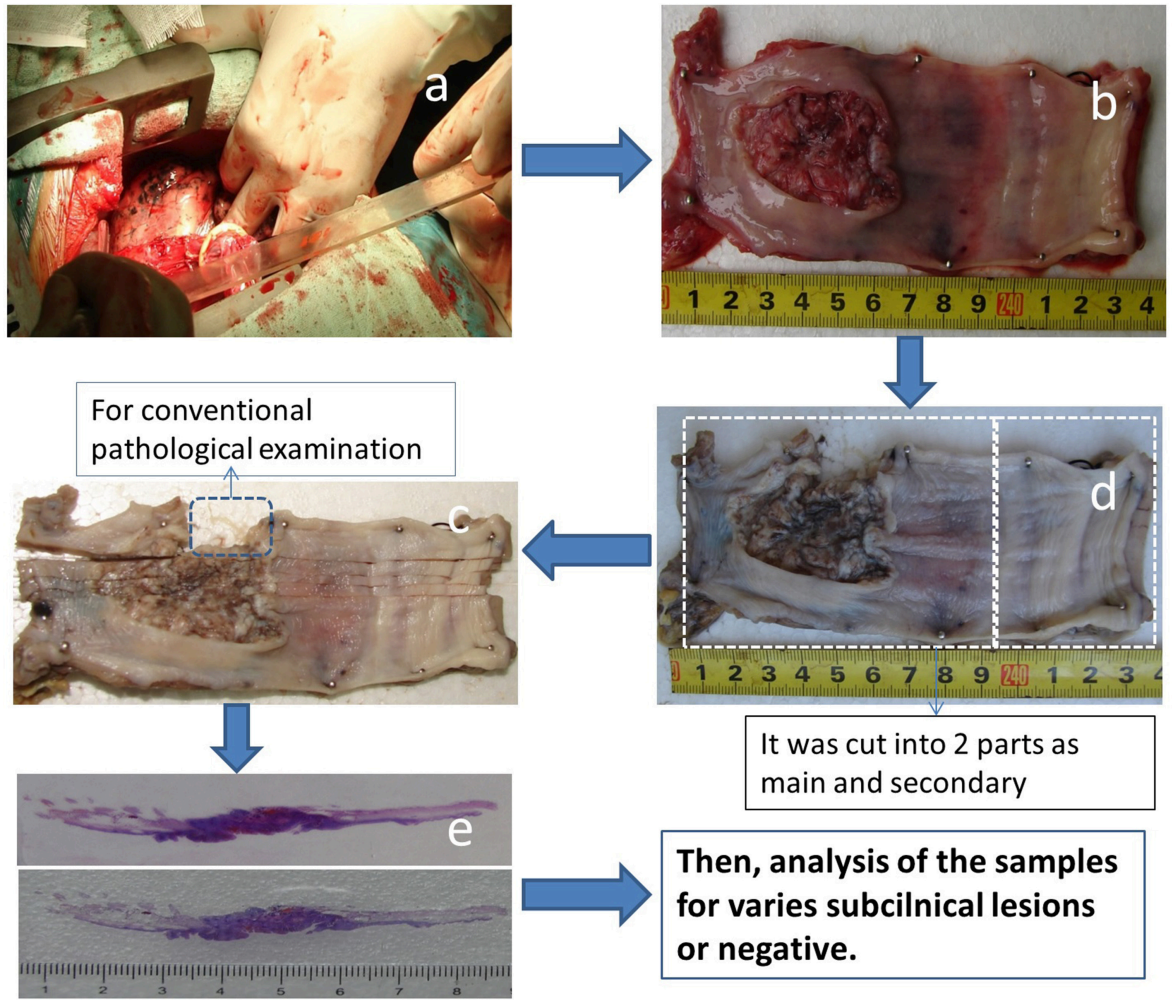
**(a–e)** The process of preparing large pathological slices.

The process for large slice preparation is shown in Figure 1. The longitudinal length of the esophagus to be resected was measured *in situ* during surgery prior to resection (Figure 1a). To correct for shrinkage of the surgical specimen, each fresh esophageal specimen was opened along the longitudinal axis after resection (with care taken not to cut through the tumor). The specimen was then carefully stretched to the same length as *in vivo*, pinned to a flat expanded polystyrene board (Figure 1b) and photographed for record keeping.

The specimen, along with the flat board, was fixed in 10% formaldehyde (Figure 1c), cut vertically into 0.5-cm-wide tissue strips that included the upper and lower cut edges (Figure 1d) and used as a large pathologic slice for examination (Figure 1e). During this step, shrinkage of the specimen was recorded after obtaining a large pathologic slice and the specimen was re-formed to its *in vivo* length.

If the length of the surgical specimen was >9 cm and ≤ 18 cm, it was cut into two parts (the main part and the secondary part), which were perpendicular to the longitudinal axis. If the specimen was >18 cm, it was cut into three parts (one main part and two secondary parts), and each part was labeled in sequence from the most cranial to the most caudal. The percent shrinkage was also calculated from the time the specimen was cut into 0.5-cm strips (Figure 1d) until the pathological examination (Figure 1e). The samples were then analyzed.

The resected lymph nodes were separately dissected and the sites were described using the nomenclature of the Japanese Society for Esophageal Diseases (11).

### Diagnostic Criteria of the Subclinical Lesions

**Figure 2:**
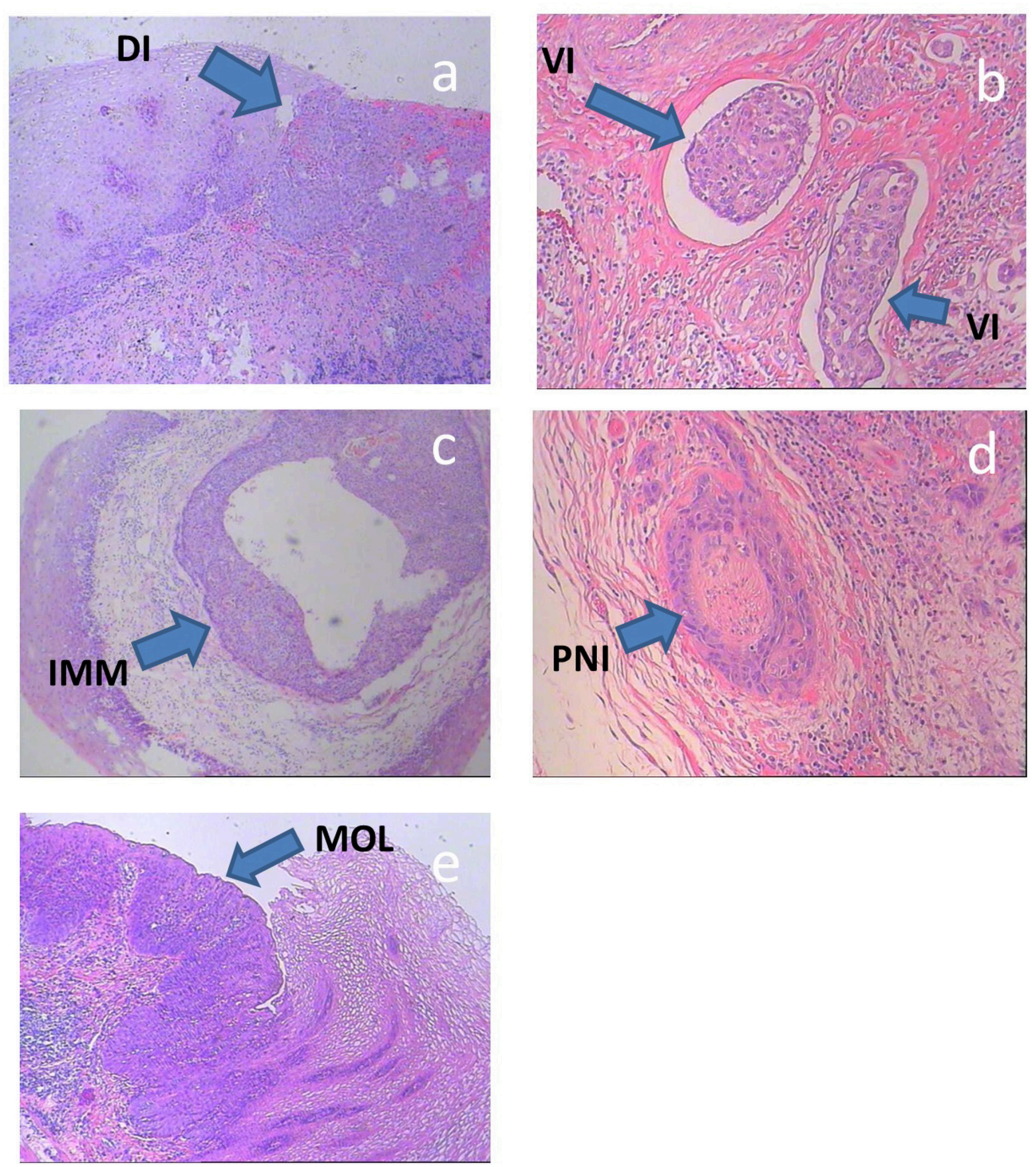
**(a–e)** Histologic specimens of typical subclinical lesions of ESCC. DI, direct invasion; IMM, intra-mural metastasis; MOL, multicentric occurrent lesions; VI, vascular invasion; PNI, perineural invasion.

The subclinical ESCC lesions were classified as: direct invasion (involving the intramucosal, submucosal, and muscular layers), intra-mural metastasis, multicentric occurrent lesions (differentiated from a second carcinoma of non-esophageal origin and other lesions, e.g., intra-mural metastasis), vascular invasion, or perineural invasion (Figure 2) (12–14).

Direct invasion involving the intramucosal, submucosal and muscular layers includes secondary lesions that infiltrate in all directions from the main tumor. In this study, direct invasion was only measured in the cranial-caudal direction, because only subclinical lesions in this direction affect the length of the CTVp.

Intra-mural metastasis, also known as salutatory metastasis, was defined in accordance with the following standard macroscopic and histologic criteria: clearly separated from the primary tumor, located in the esophageal wall, the gross appearance of a submucosal tumor without intra-epithelial extension of the tumor, of the same histological type as the primary tumor, and without evidence of intravascular growth.

Multicentric occurrent lesions must be differentiated from a second carcinoma of non-esophageal origin and other lesions. The main tumor is located in the esophagus or the gastroesophageal junction. Multicentric occurrent lesions have definite malignant features, as determined by pathological analysis, and individual lesions are discontinuous. The main tumor and secondary tumor coexist, and the main tumor is larger and more deeply invasive than the secondary tumor. The secondary tumor lesions include intraepithelial carcinomas, such as atypical hyperplasia and carcinoma *in situ*, metastatic lesions were excluded. The secondary tumors that occur after the primary esophageal carcinoma (~1 year later) are usually known as heterochronic multi-esophageal carcinoma and should not be included in the multicentric lesion category.

Vascular invasion is defined as the infiltration of tumor cells into lymph and blood vessels, including tumor embolus formation.

Perineural invasion involves cancer cell infiltration into the perineurium or fasciculus and can be detected at the boundary of the deepest tumor invasion, as well as by metastasis outside of the primary tumor site (15).

### Statistical Analysis

SPSS software (version 18.0; SPSS, Chicago, IL, USA) was used for statistical analyses. Sensitivities were compared using the chi-squared or Fisher's exact tests. A 2-dimensional logistic regression model was used to estimate the associations between the clinicopathological features and the microscopic spread of lesions.

## Results

### Subclinical Lesions and Clinicopathological Parameters

There were 55 patients with ESCC enrolled in this study. The following clinicopathological parameters were associated with the presence of subclinical lesions (according to the chi-squared and Fisher's exact tests): tumor length, differentiation, pathologic tumor status, pathologic lymph node status and stage (Table 1). The age and gender of the patient and the tumor location were not statistically related to subclinical lesions.

**Table 1:** Correlation of subclinical lesions with patients' clinicopathological parameters in esophageal SCC.

**Variables**	**Subclinical lesions**			
	**Cases**	**Absent**	**Present**	***P*-value**
Age (yrs)^¶^				0.919
≤ 57	28	10 (35.7%)	18 (64.3%)	
>57	27	10 (37.0%)	17 (63.0%)	
Sex				0.731
Male	40	14 (35.0%)	26 (65%)	
Female	15	6 (40.0%)	9 (60.0%)	
Location^*^				0.528
Upper	9	4 (44.4%)	5 (55.6%)	
Middle	22	6 (27.3%)	16 (72.7%)	
Lower	24	10 (41.7%)	14 (58.3%)	
Tumor length (cm)^§^				0.035
≤ 4.2	31	15 (48.4%)	16 (51.6%)	
>4.2	24	5 (20.8%)	19 (79.2%)	
Differentiation^*^				0.032
Well	13	9 (69.2%)	4 (30.8%)	
Moderate	28	9 (32.1%)	19 (67.9%)	
Poor	14	3 (21.4%)	11 (78.6%)	
pT status				0.000
T1-2	23	15 (65.2%)	8 (34.8%)	
T3	32	5 (15.6%)	27 (84.4%)	
pN status^*^				0.000
N0	17	13 (76.5%)	4 (23.5%)	
N+	38	7 (18.4%)	31 (81.6%)	
Stage^*^				0.000
I	7	6 (85.7%)	1 (14.3%)	
II	20	12 (60.0%)	8 (40.0%)	
III	28	2 (7.1%)	26 (92.9%)	

¶ Median age;

* compared with Fisher's exact test;

§ *Median length; T1-2, including T1 and T2 (Absent/Present ratios are 4/0 and 11/8, respectively); N+, including N1, N2, and N3 (Absent/Present ratios are 6/14, 1/12, and 0/5, respectively)*.

### Shrinkage of the Large Pathological Slice Specimens

The length of the esophagus to be resected was measured *in situ*, and again after fixation in 10% formaldehyde (Figure 1c). The mean percent shrinkage was 0.081 ± 0.041% for the 113 specimens. Fifty-two of the specimens were separated into two parts (51 specimens between 9 and 18 cm, one equal to 18 cm). Three specimens were separated into three parts (those >18 cm).

### Presence and Distance of Subclinical Lesions Beyond the Gross Tumor

Subclinical lesions were found in 63.64% (35/55) of patients (including direct invasion, intra-mural metastasis, multicentric occurrent lesions, vascular invasion, and perineural invasion; Table 2). Of the 35 ESCC patients with subclinical lesions, 3.29 ± 1.25 lesions were observed.

**Table 2:** The incidence of subclinical lesions and their distance from the main tumor (55 patients).

**Categorization**	**Number of positive patients (%)**	**Direction**	**Number of lesions**	**Maximum distance (cm)**	**Mean distance (cm)**	***SD***
DI	31 (56.37%)	Cranial	23	0.90	0.34	0.22
		Caudal	26	0.80	0.45	0.16
IMM	17 (30.90%)	Cranial	10	2.60	1.60	0.50
		Caudal	10	3.50	1.60	0.77
MOLs	22 (40.00%)	Cranial	10	8.00	2.20	2.02
		Caudal	14	2.80	1.80	0.65
VI	12 (21.80%)	Cranial	6	2.70	1.45	0.66
		Caudal	7	3.00	1.74	0.76
PNI	10 (18.20%)	Cranial	4	1.10	0.80	0.31
		Caudal	6	1.80	0.90	0.49

**Figure 3:**
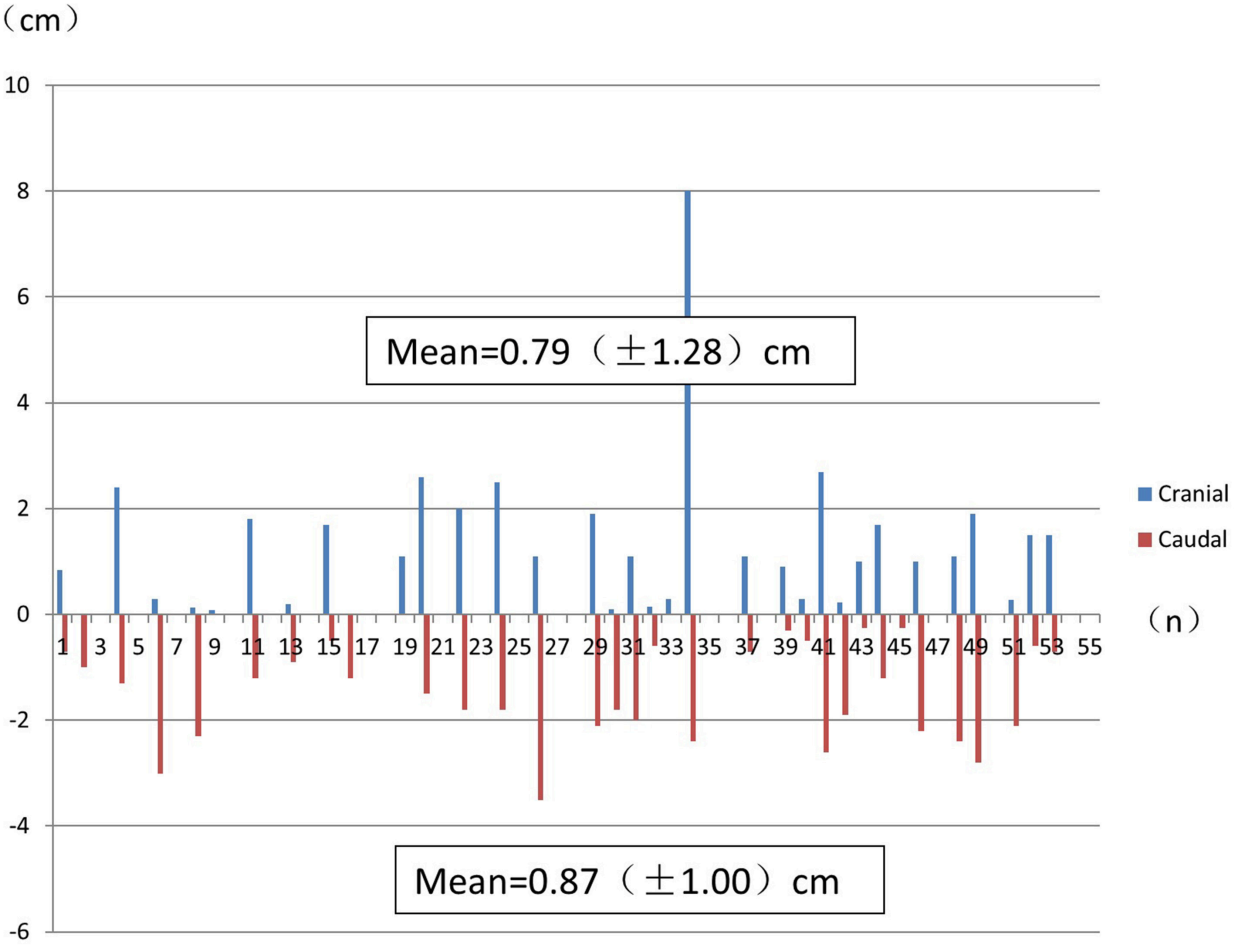
The greatest distance of subclinical lesions beyond the gross tumor for each histological specimen from each patient. Numbers indicate the mean ± SD.

Considering all the histological specimens of all patients, the greatest distances of the subclinical lesions beyond the gross tumor were 0.79 ± 1.28 cm (cranial) and 0.87 ± 1.00 cm (caudal; Figure 3).

**Figure 4:**
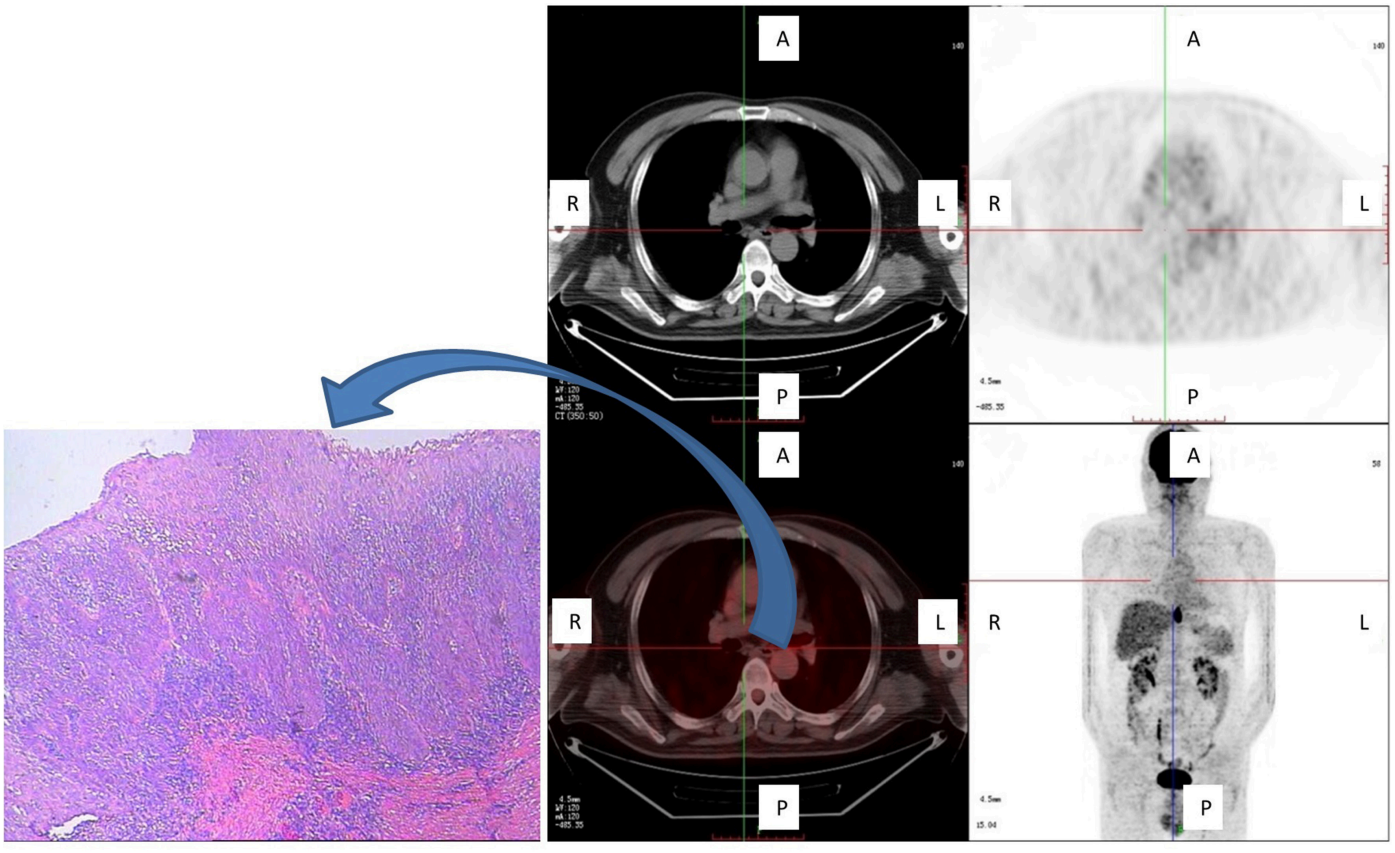
One patient with a subclinical (multicentric) lesion, which was the greatest cranial distance from the main tumor. This lesion was not detected *in situ* by FDG PET/CT, but was detected by pathological examination (carcinoma *in situ*) and was located cranial to the main tumor. Shown are axial PET, axial fused PET/CT, wholebody maximum intensity projections, and pathological specimen of secondary tumor manifestation at the plane marked by the cross-sign.

The furthest distance that a subclinical lesion was observed from the main tumor was 8.0 cm, which involved a multicentric lesion located cranial to the main tumor site in a 53-year-old male patient with middle thoracic ESCC. This lesion was not detected *in situ* by FDG PET/CT (Figure 4).

### Association Between Subclinical Lesions and Clinical Pathological Parameters

The number of subclinical lesions correlated with the gross length of the tumor (*R* = 0.356, 95% CI = 0.028–0.667, *P* = 0.035) and with the FDG PET/CT SUVmax (*R* = 0.487, 95% CI = 0.119–0.689, *P* = 0.003; Table 3). Additionally, there was a trend in association between the SUVmax values of the primary tumor and the number of subclinical lesions. In addition, the number of subclinical lesions correlated with the MTV (*R* = 0.342, 95% CI = −0.099–0.661, *P* = 0.044).

**Table 3:** SUVmax, determined by FDG PET/CT, of patients with subclinical lesions.

**No**.	**Number of subclinical lesions**	**Length of gross tumor (cm)**	**SUVmax**	**MTV (ml)**
1	2	3.5	5.4	7.72
2	3	5.3	13.6	25.93
3	4	4.0	11.0	17.19
4	2	2.7	9.5	7.12
5	4	2.9	18.6	8.69
6	1	4.3	10.5	22.98
7	4	5.9	18.2	34.46
8	3	2.6	8.8	18.40
9	3	2.0	8.5	22.93
10	2	5.2	13.0	24.76
11	2	4.2	10.2	12.37
12	2	3.7	11.0	19.56
13	5	8.8	13.1	67.50
14	2	8.0	23.0	119.20
15	4	6.8	11.1	20.30
16	5	7.0	14.7	55.70
17	4	6.0	11.0	25.00
18	4	6.0	18.2	30.00
19	3	4.2	13.3	13.10
20	1	4.1	7.8	12.50
21	4	3.5	16.0	12.40
22	4	7.0	15.8	54.90
23	4	4.2	12.0	12.88
24	3	9.3	20.0	76.85
25	6	10.0	63.8	150.50
26	4	4.2	11.6	28.10
27	2	4.0	13.5	24.87
28	5	5.5	15.8	27.80
29	1	4.8	11.2	22.13
30	3	3.0	7.3	15.50
31	3	4.7	13.0	24.80
32	5	4.0	20.5	32.50
33	4	4.6	13.7	37.60
34	4	6.5	8.1	33.10
35	3	5.5	9.5	30.90

## Discussion

An objective of this study was to clarify the most appropriate CTVp margins in patients with ESCC. To determine this distance and its correspondence with FDG PET/CT results, we employed large pathological slices that included the entire tumor and the resected proximal tissue. This long and complex process provided an intact specimen in which the anatomical relationship between the tumor and the surrounding tissue can be observed, which was essential for assessing the presence of lesions.

Using various pathological techniques, previous studies have reported different rates of subclinical lesion occurrence. However, there has not been a comprehensive report that evaluated all types of subclinical lesions that make up the CTV in esophageal cancer, and the CTVp range is still controversial. Although there have been several studies addressing this, they seldom used large pathological slices (12, 16–18).

Kuwano et al. (19) reported that the mean distance of direct invasion from the main tumor was 4.11 mm (range: 1.2–9.5 mm). However, according to Tsutsui et al. (20), direct invasion was usually < 30 mm. In our study, direct invasion was observed in 56.37% of the patients (cranial and caudal) and the greatest distance was 9.5 mm. Other studies have reported finding intra-mural metastasis in 4.19–26.0% of patients, and the distance of intra-mural metastasis from the primary tumor ranged from 1–130 mm, with maximum cranial and caudal distances of 130 mm and 95 mm, respectively (21–24). Our research results indicated that 30.90% of patients had intra-mural metastasis, with a maximum distance of 35.0 mm.

Several studies have reported that the incidence of multicentric occurrent lesions ranges from 20.20 to 31.00% in patients who did not receive preoperative irradiation, with a cranial distance from the primary tumor of 0.88–7.14 cm and a caudal distance of 0.57–6.26 cm (10, 12). In our study, the incidence of multicentric occurrent lesions was 40.00%, and the greatest distance that a lesion was observed was 8.0 cm in the cranial direction.

Several clinical trials have shown that preoperative radiation can reduce the incidence of multicentric occurrent lesions. For example, Kuwano et al. (12) reported that multicentric occurrent lesions were found in 11.70% (19/162) of patients who received preoperative irradiation and in 25.60% (11/143) of those who did not. In addition, Tsutsui et al. (20) reported that only 5.61% (17/303) of patients had multicentric occurrent lesions, most of whom were administered preoperative irradiation.

The Japanese researchers Lam et al. (17) observed vascular invasion in 16.67% (16/96) of ESCC patients, mostly at the base of the tumor, and occasionally distal to the primary tumor, at a maximum distance of 5 cm. Vascular invasion has been putatively associated with advanced tumor stages, with rates of 13.89% (15/108) in the early stage (25) to 39.10% (143/366) in the advanced stage (26). In our study, vascular invasion was found in 21.80% of the study population, and the greatest distance was 3.00 cm in the caudal direction.

Sarbia et al. (27) first reported perineural invasion in 26.10% (42/161) of patients with ESCC in 1995. Through univariate and multivariate survival analyses, the authors concluded that perineural invasion was not a prognostic factor. In our study, perineural invasion was present in 18.20% of patients, and the greatest distance was 1.80 cm in the caudal direction. Conversely, Tanaka et al. (28) reported perineural invasion in 46.20% (48/104) of resected ESCCs and concluded that perineural invasion is an important prognostic factor for local relapse. Recently, Chen et al. (29) also reported that perineural invasion is a prognostic factor for ESCC and suggested that perineural invasion status should be considered when planning therapy strategies.

Kato et al. (30) reported that it is difficult to detect lymph node metastases of 0.6 to 0.8-cm size with FDG PET/CT. In our study, lesions with a diameter < 0.5 cm could not be detected using FDG PET/CT. Furthermore, subclinical lesions of several microns could not be detected using FDG PET/CT (Figure 3).

Even with today's advanced imaging technology, some subclinical lesions cannot be detected directly. In this study, there was a trend toward higher FDG PET/CT SUVmax and MTV values associating with the presence of subclinical lesions. SUVmax correlated with the number of lesions (*R* = 0.487, 95% CI = 0.119–0.689, *P* = 0.003), as did the MTV (*R* = 0.342, 95% CI = −0.099–0.661, *P* = 0.044). As imaging technology advances, the ability to detect smaller cancer foci may improve.

Regardless of the controversy concerning CTVp data, clinical experience has shown that enlarged radiation fields do not improve local control rates or overall survival, despite the extremely toxic nature of radiation (31). The extent of subclinical lesions exceeds the conventional extent of the margins, as the results of the current research indicate. In many authors' views, it is also a good choice to employ involved-field irradiation; therefore, radiation-related toxicity would decline in cases where the target volume was diminished.

In theory, the treatment of subclinical lesions may benefit from lower doses of radiation. A total dosage of 50 Gy administered in 2-Gy fractions is effective for achieving an overall 90% reduction in subclinical metastases, and the cancer cell burden in some patients can be wiped out by these low doses. Thus, significant rates of disease control can still be achieved when patient tolerance suggests lower-than-optimal doses, as shown by the linear association and absence of a significant threshold in the dose-response curve. Small lesions can be treated by low doses of radiation (autoimmune extermination), but the optimal dose for this treatment still needs further study.

In our study, we concluded that the SUVmax and MTV obtained via FDG PET/CT may be able to indicate the existence of subclinical lesions. Imaging guided by field involvement will benefit more patients with ESCC in terms of dose-limitation for the organs at risk, such as the lungs, heart and spinal cord (32).

Because our sample size was limited, our analysis did not include all variables associated with subclinical lesions. The safe surgical margin for primary ESCC tumors classified as Tis (high-grade dysplasia) or T1 (invasion of lamina propria, muscularis mucosae, or submucosa) is usually 1 cm in most endoscopic mucosal resections and dissections (33). However, for T2 (invasion of the muscularis propria) and more aggressive diseases, the safe margin is uncertain, and studies with a larger sample size are needed.

Secondary lesions with diameters from several microns to 2 mm, as well as multicentric occurrent lesions, are more likely to occur in patients with certain risk factors. These include male gender, heavy drinking or smoking, and a family history of carcinoma of the upper digestive tract (11). The CTVp margin should be enlarged appropriately if these risk factors are present.

To correct for the shrinkage of the resected esophagus, we stretched the surgical specimen to the same length as *in situ* by pinning it to a board prior to fixing it with formaldehyde. The amount of shrinkage was low; however, other authors have reported greater degrees of shrinkage. For example, Siu et al. (34) reported that shrinkage was usually 50% of the *in situ* length.

## Conclusions

Our results suggest that to cover 94.5% of subclinical lesions in the CTVp of ESCC, a 3-cm margin in the cranial-caudal direction should be added to the primary gross tumor volume. Both the SUVmax and MTV obtained through FDG PET/CT may predict subclinical lesions, although the imaging did not detect subclinical lesions directly. By our findings, it may be safe to use involved-field radiation for ESCC, although validation of this should be sought through future prospective and randomized studies.

## Ethics Statement

All patients gave their written informed consent in accordance with the Code of Ethics of the World Medical Association (Declaration of Helsinki) for experiments.

Shandong University Affiliated Shandong Cancer Hospital Ethics Committee allows the use of patient data for research, provided that any person's related data are kept anonymous. The Ethics Approval authorization is SDTHEC201103009, approved on 5 March 2011.

## Author Contributions

JY and DH designed the experiments. YY, JC, AR, PS, and ZF carried out experiments and data collection. GZ, LW, and DH analyzed experimental results and developed analysis tools. DH wrote and reviewed the manuscript. All authors read and approved the final manuscript.

### Conflict of Interest Statement

The authors declare that the research was conducted in the absence of any commercial or financial relationships that could be construed as a potential conflict of interest.

## Abbreviations

CT: computed tomography
CTVp: primary clinical target volume
ESCC: esophageal squamous cell carcinoma
FDG: ^18^F-fluorodeoxyglucose
MTV: metabolic tumor volume
PET: positron emission tomography
SUV: standardized uptake value.
